# Excited States of Nucleic Acids Probed by Proton Relaxation Dispersion NMR Spectroscopy

**DOI:** 10.1002/anie.201605870

**Published:** 2016-08-17

**Authors:** Michael Andreas Juen, Christoph Hermann Wunderlich, Felix Nußbaumer, Martin Tollinger, Georg Kontaxis, Robert Konrat, D. Flemming Hansen, Christoph Kreutz

**Affiliations:** ^1^ Institute of Organic Chemistry and Center for Molecular Biosciences Innsbruck (CMBI)University of InnsbruckInnrain 80/826020InnsbruckAustria; ^2^ Bachem Americas, Inc.3132 Kashiwa StreetTorranceCA 90505USA; ^3^ Computational Biology and Biomolecular NMRMax F. Perutz Laboratories (MFPL)University of ViennaDr. Bohr Gasse 9 (VBC 5)1030ViennaAustria; ^4^ Institute of Structural and Molecular BiologyDivision of BiosciencesUniversity College LondonDarwin Building, Room 612, Gower StreetLondonWC1E 6BTUK

**Keywords:** DNA, NMR spectroscopy, proton relaxation dispersion, RNA, stable isotope labeling

## Abstract

In this work an improved stable isotope labeling protocol for nucleic acids is introduced. The novel building blocks eliminate/minimize homonuclear ^13^C and ^1^H scalar couplings thus allowing proton relaxation dispersion (RD) experiments to report accurately on the chemical exchange of nucleic acids. Using site‐specific ^2^H and ^13^C labeling, spin topologies are introduced into DNA and RNA that make ^1^H relaxation dispersion experiments applicable in a straightforward manner. The novel RNA/DNA building blocks were successfully incorporated into two nucleic acids. The A‐site RNA was previously shown to undergo a two site exchange process in the micro‐ to millisecond time regime. Using proton relaxation dispersion experiments the exchange parameters determined earlier could be recapitulated, thus validating the proposed approach. We further investigated the dynamics of the cTAR DNA, a DNA transcript that is involved in the viral replication cycle of HIV‐1. Again, an exchange process could be characterized and quantified. This shows the general applicablility of the novel labeling scheme for ^1^H RD experiments of nucleic acids.

Conformational heterogeneity expands the functional repertoire of nucleic acids.^1^ These transitions between structurally different conformational states add an additional layer of functional adaptability of nucleic acids. NMR spectroscopy has proven to be very well suited to investigate such transitions and to detected and characterize so called excited states.1e, 2 These low populated sub‐states represent high free‐energy conformations, that are speculated to be involved in important processes, such as ligand binding, protein–nucleic acid recognition or catalysis by ribozymes. Several methods, like chemical exchange saturation transfer (CEST), *R*_1ρ_ and CPMG relaxation dispersion experiments, have been recently applied to nucleic acids and have proven to be suitable to characterize kinetic, thermodynamic, and structural parameters of the exchange process between the ground and excited state.^3^ Noteworthy, a ^1^H CEST experiment was recently introduced by Dayie and co‐workers. With the aid of this experiment proton chemical shifts of the *S*‐adenosylmethionine (SAM) II riboswitch excited state could be determined. In their work a slow exchange process (*k*_ex_≈30 s^−1^) was found with the high energy state conformation resembling the SAM bound state supporting a conformational selection mechanism for this aptamer. The approach also benefited from site‐specific ^13^C‐1′‐, ^13^C‐6‐labeling resulting in the absence of strong ^1^ *J*_CC_‐couplings.3h

Typically, experiments to address functional dynamics obtain the parameters from X‐spin relaxation data (X being ^13^C or ^15^N). It would be very beneficial to adapt NMR methods and isotope labeling protocols for nucleic acids to apply RD experiments on proton spins, as the experimental constraints are less stringent for ^1^H as discussed below. This method has been successfully applied to proteins only and has provided insights into for example, functional dynamics during protein folding and enzyme catalysis.^4^ Several advantages make ^1^H RD experiments very attractive for the characterization of excited states. For example, reduced sample heating during the CPMG train, a shorter 180° pulse length, and a wider range of the effective CPMG field strengths (up to 5 kHz) can extend the CPMG method to address transient states with shorter lifetimes. Even more important, the ^1^H RD experiment would give access to excited‐state proton chemical shifts. Recently, ^1^H chemical shifts were shown to be very sensitive reporters of nucleic acid tertiary structure and were used for the de novo 3D structure prediction of RNA.^5^ Thus, ^1^H RD experiments create the opportunity to use excited state proton chemical shifts to obtain insights into structural details of the low populated high‐energy conformation.

One mandatory prerequisite to apply the ^1^H RD NMR experiment is the absence of large homonuclear ^1^H–^1^H scalar couplings. In nucleic acids the low proton density in the nucleobase moieties facilitates the applicability of the experiment. For example, purine nucleobases offer per se an ideal spin topology for proton RD experiments. The absence of nearby protons makes the ^1^H–C8 spin pair of adenine and guanine ideally suited for ^1^H RD experiments. For the simple and selective read‐out of the H8 resonance, ^13^C labeling of position 8 in purines would be advantageous. Thus, the synthetic access to 8‐^13^C‐purine DNA and RNA phosphoramidites and the incorporation into a nucleic acid via the solid phase synthesis approach (Figure 1 a and b) allows for conducting ^1^H relaxation dispersion experiments in nucleic acids. Experimental details on the synthesis of 8‐^13^C‐RNA purine amidites can be found in the supporting information. The synthesis of the 8‐^13^C‐purine DNA amidites will be published elsewhere.

**Figure 1:**
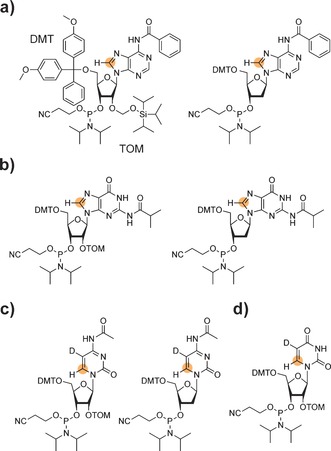
Next generation stable isotope labeled RNA and DNA phosphoramidites. a) Adenosine RNA (left) and DNA (right) phosphoramidite with position 8 labeled with carbon‐13. b) Guanosine RNA (left) and DNA (right) phosphoramidite with position 8 labeled with carbon‐13. c) Cytidine RNA (left) and DNA (right) phosphoramidite with position 6 labeled with carbon‐13 and H5 replaced by deuterium. d) 6‐^13^C‐5‐d‐Uridine RNA phosphoramidite. Abbreviations: DMT 4,4′‐Dimethoxytrityl, TOM Triisopropylsilyloxymethyl, D Deuterium. Orange circle=^13^C label.

We earlier reported on the synthesis of 6‐^13^C‐pyrimidine RNA phosphoramidites.3d The uracil and cytosine nucleobases do not offer an appropriate spin topology due to presence of the H5/H6 scalar coupling (^3^ *J*_H5/H6_≈8 Hz). This issue can be circumvented by the inclusion of a selective deuteration step to replace the proton at position 5 by deuterium yielding 6‐^13^C‐5‐d‐pyrimidine building blocks with a very small D5/H6 scalar coupling constant (^3^ *J*_D5/H6_≈1 Hz) making ^1^H RD experiments feasible even in the absence of deuterium decoupling (Figure 1 c and d). The synthesis of the 6‐^13^C‐pyrimidine RNA building blocks was described in detail earlier.3d The additional deuteration step is now included in the supporting information. The synthesis of the 6‐^13^C‐5‐d‐2′‐deoxycytidine amidite will be reported elsewhere.

Using the novel building blocks 13 nucleotides of the 27 nt A‐site mimic were replaced with their ^13^C/^2^H labeled counterparts (Figure 2 a). The successful incorporation was confirmed by mass spectrometry (Figure S1 a in the Supporting Information) and NMR spectroscopy (Figure 2 b).

**Figure 2:**
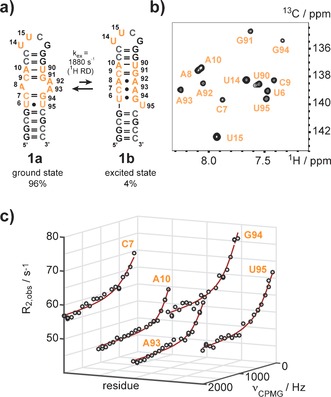
Excited state of the A‐site RNA probed by ^1^H relaxation dispersion NMR. a) The 27 nt A‐site RNA mimic switches between two alternate secondary structures. R_1_ρ RD experiments conducted earlier gave an exchange rate of 4 kHz and an excited state population of 2.5 %.3a The ^13^C and/or ^2^H modified residues are highlighted in orange. b) ^1^H‐^13^C‐HSQC spectrum of site‐specifically isotope modified A‐site RNA. The spectrum is in accordance with earlier published data. c) Selected non‐flat ^1^H relaxation dispersion profiles of residues C7, A10, A93, G94 and U95 acquired at 600 MHz resonance frequency and 12 °C. The black open circles represent the experimental data, the red line the best fit. A global fit gave an exchange rate of 1880±140 s^−1^ and an excited state population of 4.06±1.28 % at 12 °C, which is in good agreement with the R_1ρ_ dispersion data obtained at room temperature.

A detailed investigation using ^13^C R_1ρ_ relaxation dispersion NMR was recently conducted on this RNA.3a The RNA is involved in the selection process of cognate and near‐cognate tRNAs in the ribosome. The residues A92 an A93 play an important role in that recognition step, as they stabilize the cognate tRNA/mRNA complex while in a flipped out state. A two state equilibrium between two secondary structures **1 a** and **1 b**—the ground state **1 a** being the functional state, the excited state **1 b** being non‐functional with A92 and 93 sequestered in non‐canonical base pairs—with an exchange rate constant of 4 kHz at 25 °C and an excited state population of 2.5 % was found. We choose this system as it is ideally suited to validate the novel isotope labeling scheme and because of its applicability to conduct proton relaxation dispersion NMR experiments. The stable isotope labeled residues were introduced around the A8–A92–A93 bulge and in the UUCG tetraloop. The bulge sequence partition is restructured in the excited state **1 b** leading to observable relaxation dispersion profiles so far shown for carbon‐13, whereas the *UU*CG loop residues are expected to show flat dispersion profiles. Based on a published proton relaxation dispersion experiment for ^15^NH spin pairs in per‐deuterated proteins we could establish a simple pulse scheme as the optimized ^13^C–^1^H spin topology in both, purine and pyrimidine nucleobases, results in the absence of large homonuclear scalar couplings and thus making the application of the ^1^H dispersion experiment very straightforward.4a,4d, 6

The pulse scheme is included in the supporting information section and is available upon request. Proton relaxation dispersion experiments were conducted at 500 and 600 MHz resonance frequencies for purines and pyrimidines and at 800 MHz for an 8‐^13^C‐adenosine labeled sample only. A selection of experimentally obtained non‐flat proton relaxation dispersion profiles is shown for the residues C7, A10, A93, G94 and U95 (Figure 2 c). Other residues, which could potentially sense the exchange process between the alternative secondary structures—such as residues U6, A8 and U90—gave no statistically significant relaxation dispersion profiles, very likely due to small chemical shift differences between ground and excited state. The loop U residues U14 and U15 exhibited flat dispersion profiles as expected. The parameters obtained—an overall exchange rate constant *k*_ex_ of 1880±140 s^−1^ and an excited state population *p*_B_ of 4.06±1.28 % at 12 °C—from fitting the ^1^H RD profiles of C7, C9, A10, A93, G94 and U95 are in good agreement with the previously reported data from ^13^C relaxation experiments (*k*_ex_=4 kHz, *p*_B_=2.5 % at 25 °C) validating the ^1^H RD approach to study milli‐ to microsecond dynamics in RNA. A summary of the exchange parameters obtained from the ^1^H relaxation data is given below (Table 1).5c We also compared the obtained chemical shift differences from the proton RD experiment with reference data for the excited state **2 b** with mutants mimicking the excited state (ΔU95 and N^3^MU95 mutants). A fairly good correlation was observed indicating that the proposed base pair reshuffling process is recapitulated via the proton RD experiment (Table S1 in the Supporting Information).

**Table 1:** Summary of exchange parameters obtained from ^1^H CPMG relaxation dispersion profiles.

	Residue	*k*_ex_ [s^−1^]^[a]^	*p*_B_ [%]^[b]^	|Δ$\bar{\omega }$ |[ppm]^[c]^
A‐site RNA **1 a**/**b** 285 K	C7 C9 A10 A92 A93 G94 U95	1880±140	4.06±1.28	0.24±0.07 0.15±0.08 0.22±0.05 0.14±0.07 0.24±0.05 0.34±0.07 0.29±0.06
				
cTAR DNA **2 a**/**b** 298 K	G4 A5 C19 G20 A21 C22 C23	525±59	1.0±0.1	0.25±0.02 0.49±0.03 0.22±0.01 0.20±0.02 0.56±0.03 0.34±0.02 0.24±0.01

[a] Exchange rate constant and [b] population from global fit of relaxation dispersion data of residues with significant relaxation dispersion at all magnetic fields. [c] Residue‐specific chemical shift difference between ground and excited state. Details on the fitting procedure are given in the Supporting Information.

We then turned our attention to the selective labeling of DNA and to study excited states in DNA using the ^1^H RD experiment. We picked the cTAR DNA (MAL isolate) **2 a**/**2 b** as a target (Figure 3 a).^7^ This DNA is involved in the viral replication cycle of the HIV‐1 and fulfils a priming function via hybridization to the viral genomic RNA. The nucleocapsid protein NCp 7 interacts with the cTAR element and the 55 amino acid polypeptide acts as a nucleic acid chaperone facilitating the DNA/RNA hybridization step.^8^ The DNA comprises 26 nucleotides and is supposed to fold into a hairpin‐bulge conformation (Figure 3 a). The previously proposed secondary structure **2 b**—an extended hairpin with an internal bulge—represents a minor conformation under the NMR conditions at ambient temperature, as the lower stem does not exhibit any observable imino proton resonances. The upper stem is formed as the signals from hydrogen bonded protons can be observed. These imino proton assignments have been published earlier.7a The assignment was further supported by selective ^15^N‐deoxy guanosine labeling (Figure S2 a and b in the Supporting Information). Thus, the major fold **2 a** was assigned to a short hairpin structure with 5′‐ and 3′‐dangling ends. We replaced 10 residues by their ^13^C/^2^H labeled counterparts and again the isotope labeling pattern was confirmed by NMR and MS (Figure 3 b and Figure S1 b in the Supporting Information). At this point we speculated that the fully folded state **2 b** is only sparsely populated. To test this hypothesis we used the proton relaxation dispersion experiment to probe milli‐ and microsecond dynamics in this DNA. We again obtained high quality data and found an exchange process for residues G4, A5, G6, C19, G20, A21, C22 and C23, which cluster in the bulge and the lower stem region and at the lower end of the upper stem (Figure 3 c). A global fit of the proton dispersion data gave an excited state population *p*_B_ of 1.0±0.1 % and an exchange rate *k*_ex_ of ca. 525±59 s^−1^. Again, a summary of the exchange parameters obtained from the ^1^H relaxation data is given below (Table 1). The dispersion data clearly confirms an exchange process located in the 5′‐ and 3′‐dangling ends. The excited state chemical shifts correlate well with standard B‐form DNA H6/H8 resonance frequencies inline with state **2 a** being more structured than ground state **1 a** (Table S1 in the Supporting Information). Using the previously reported mutate and chemical shift fingerprint (MCSF) approach we currently investigate the secondary structure of the cTAR DNA excited state in more detail and the influence of the nucleocapsid protein NCp 7 on the exchange process.3a

**Figure 3:**
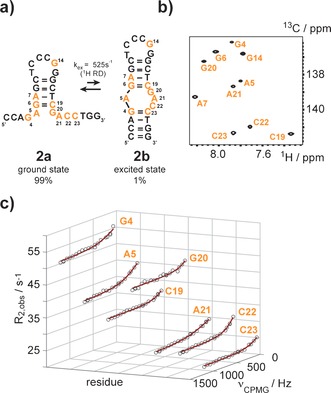
Excited state of the cTAR DNA probed by ^1^H relaxation dispersion NMR. a) The 26 nt cTAR DNA fluctuates between state **2 a** and a fully folded state **2 b**. The ^13^C and/or ^2^H modified residues are highlighted in orange. b) ^1^H‐^13^C‐HSQC spectrum of site‐specifically isotope modified cTAR DNA. c) Non flat ^1^H relaxation dispersion profiles of residues G4, A5, C19, G20, A21, C22 and C23 acquired at 600 MHz proton resonance frequency and 25 °C.The black open circles represent the experimental data, the red line the best fit. A global fit gave an exchange rate of 525±59 s^−1^ and an excited state population of 1.0±0.1 %.

Taken together, we have introduced a stable isotope labeling protocol for nucleic acids that allows for the first time the extraction of accurate chemical exchange parameters from proton relaxation dispersion experiments. The dispersion experiment can be used to characterize dynamic processes occurring at the ms‐ to μs‐time scale. Furthermore, it has been recently shown that proton chemical shifts are sensitive reporters of RNA tertiary structure and that the ^1^H chemical shift values can be used as input parameters for the de novo high resolution structure prediction using CS Rosetta RNA.5c Our NMR based approach yields absolute value proton chemical shift differences between the ground and the excited state. Combining this shift difference data from the ^1^H dispersion profiles with NMR experiments that allow the determination of the sign of the chemical shift difference opens the possibility to reconstruct the nucleobase's proton spectrum of the excited state.^9^ A method to utilize this sparse proton chemical shift data together with residual dipolar couplings (RDCs) in the structure elucidation of excited states is currently developed in our laboratories.

We also plan to transfer the revised stable isotope labeling pattern to ribonucleotide triphosphates, which can be used in the enzymatic production of RNA.3i, 10 This will significantly contribute to the dissemination of the presented approach, as T7 RNA polymerase in vitro transcription is the most widespread methodology to produce stable isotope modified RNA for NMR spectroscopy.

## Supporting information

As a service to our authors and readers, this journal provides supporting information supplied by the authors. Such materials are peer reviewed and may be re‐organized for online delivery, but are not copy‐edited or typeset. Technical support issues arising from supporting information (other than missing files) should be addressed to the authors.

Supplementary
