# Supplementary material for: Excited States of Nucleic Acids Probed by Proton Relaxation Dispersion NMR Spectroscopy
Source: Angew Chem Int Ed Engl. 2016 Aug 17;55(39):12008–12. doi: 10.1002/anie.201605870 (PMC5082494; doi:10.1002/anie.201605870)
Supplement: Supplementary file 1 — Supplementary [file ANIE-55-12008-s001.pdf]

Angewandte Chemie
Eine Zeitschrift der Gesellschaft Deutscher Chemiker

Supporting Information

Excited States of Nucleic Acids Probed by Proton Relaxation Dispersion NMR Spectroscopy

Michael Andreas Juen+, Christoph Hermann Wunderlich+, Felix Nußbaumer, Martin Tollinger, Georg Kontaxis, Robert Konrat, D. Flemming Hansen, and Christoph Kreutz**

anie_201605870_sm_miscellaneous_information.pdf

EXPERIMENTAL SECTION

General. NMR spectra were acquired on a Varian 500 MHz DD2 instrument or a Bruker 300 MHz Avance instrument. Chemical Shifts are reported relative to TMS and referenced to residual proton solvent signal: $CDCl_3$ (7.26 ppm) for ^1H NMR spectra and $CDCl_3$ (77.0 ppm) for ^{13}C spectra. DMSO-d_6 (2.50 ppm) for ^1H NMR spectra and DMSO-d_6 (39.52 ppm) for ^{13}C spectra. D_2O (4.79 ppm) for ^1H NMR spectra. ^{31}P shifts are reported relative to external phosphoric acid (85 %). ^1H assignments are based on gradient selected COSY experiments. ^{13}C shifts are assigned from a gradient selected phase sensitive HSQC and magnitude HMBC experiments. Silica 60F-254 plates were used for TLC (thin layer chromatography). For FC (flash column chromatography) silica gel 60 (230-400 mesh) was used. Reagents and solvents were purchased from Sigma-Aldrich and used without further purification. Organic solvents were extensively dried using freshly activated molecular sieves (4 Å).

Synthesis of 8-^{13}C-purine RNA phosphoramidites

Synthesis of N^6-Benzoyl-8-^{13}C-adenine (S2)

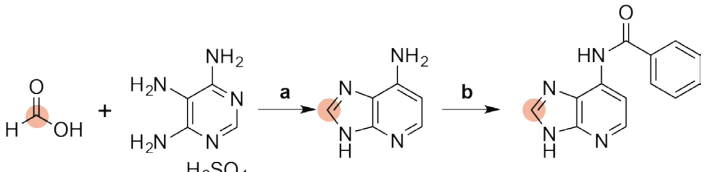

Supporting Scheme 1. Synthesis of N^6-Benzoyl-8-^{13}C-adenine **S2**. Reaction conditions: **a** morpholine, 3 h, 100-200 °C, 64%. **b** benzoylchloride in pyridine, 3 h, 135 °C, 86%. Orange cirlce = ^{13}C.

8-^{13}C-Adenine (S1)

Morpholine (1.93 g, 22.2 mmol) was cooled to 0°C and then ^{13}C-formic acid (855 µl, 22.2 mmol) was added dropwise. In a vigorous reaction morpholinium formiate was formed, which crystallized after a few minutes. 4,5,6-Triaminopyrimidine sulfate (1.65 g, 7.4 mmol) was added to the morpholinium formiate and the mixture was heated to 100°C for 1 h. Then, the temperature was raised to 200°C and heating was continued for 2h. Then, the reaction mixture was cooled to room temperature and ethanol was added. The suspension was refluxed for 30 min at 100°C and the precipitate was filtered off. The solid was transferred to a round bottom flask and dissolved in water. The solution was treated with 2 ml concentrated aqueous ammonia (28%) and then concentrated until a precipitate was formed. The suspension was stored overnight at 4°C. 8-^{13}C-Adenine was isolated by filtration and dried in high vacuum.

Yield: 644 mg (4.7 mmol, 64%)
^{1}H-NMR (300 MHz, DMSO-d_6, 25°C)
δ 12.84 (s, 1H, N*H*); 8.11 (s, 1H, C(2)*H*); 8.08 (d, 1H, C(8)*H*, $^1J_{CH}$ = 210 Hz); 7.06 (s, 2H, NH_2) ppm.
^{13}C-NMR (75 MHz, DMSO-d_6, 25°C)
δ 152.38 (*C*(2)H); 138.85 (*C*(8)H) ppm.

N^6-Benzoyl-8-^{13}C-Adenine (S2)

8-^{13}C-Adenine **S1** (500 mg, 3.7 mmol) was suspended in anhydrous pyridine (12 ml) and benzoyl chloride (508 µl, 4.4 mmol) was ded under stirring at room temperature. The reaction mixture was stirred at 135°C for 3 h. After cooling to room temperature pyridine was removed by evaporation and the residue was co-evaporated twice with toluene. The residual oil was suspended in saturated sodium bicarbonate solution. After the CO_2 evolution has ceased, the precipitate was filtered off, washed with chloroform and dried in high vacuum.

Yield: 755 mg (3.2 mmol, 86%)
^{1}H-NMR (300 MHz, DMSO-d_6, 25°C)

δ 12.03 (s, 2H, N*H*); 8.72 (s, 1H, C(2)*H*); 8.48 (d, 1H, C(8)*H*, $^1J_{CH}$ = 211 Hz); 8.10 (s, 2H, C(arom)*H*); 7.69 (m, 2H, C(arom)*H*); 7.54 (m, 2H, C(arom) *H*) ppm.
<u>^{13}C-NMR (75 MHz, DMSO-d_6, 25°C)</u>
δ 150.97 (*C*(2)H); 145.97 (*C*(8)H); 128.47 (*C*(arom)H) ppm.

Synthesis of N^6-Benzoyl-5'-O-DMT-2'-O-TOM-8-^{13}C-adenosine 3'-O-CEP (S7)

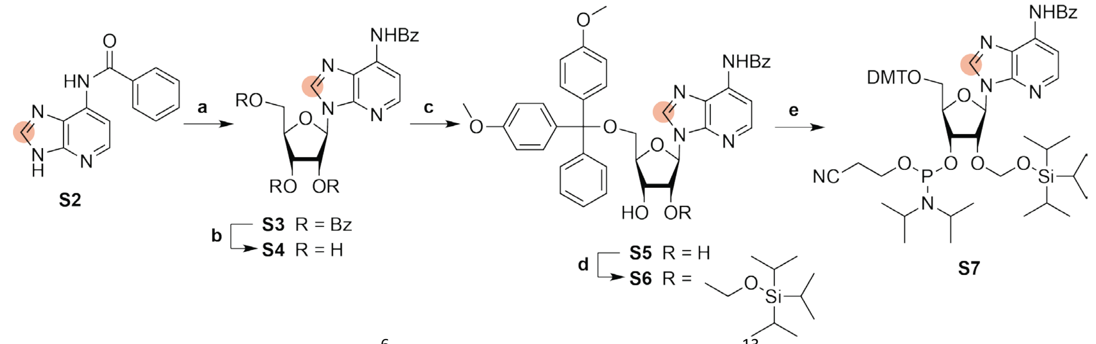

Supporting Scheme 2. Synthesis of N^6-Benzoyl-5'-*O*-DMT-2'-*O*-TOM-8-^{13}C-adenosine 3'-*O*-CEP **S7**. Reaction conditions: **a** 1-*O*-Acetyl-2,3,5-tri-*O*-benzoyl-β-*D*-ribofuranose (ATBR), *N,O*-Bis(trimethylsilyl)acetamide (BSA), trimethylsily trifluoromethanesulfonate (TMSOTf) in toluene, 1 h, 105°C, 75%. **b** 1N NaOH in pyridine/ethanol 1/1, 30 min, 0°C, 100%. **c** 4,4'-Dimethoxytrityl chloride (DMT-Cl) in pyridine, 4 h, rt, 74%. **d** Di-tert.-butyltin dichloride, *N.N*-diisopropylethylamine (DiPEA), triisopropylsilyloxymethyl chloride (TOM-Cl), 1 h, 80°C, 49%. **e** 2-Cyanoethyl *N,N*-diisopropylchlorophosphoramidite (CEP-Cl), DiPEA in methylene chloride, 2 h, rt, 72%. Orange cirlce = ^{13}C.

N^6-Benzoyl-5',3',2'-tri-*O*-benzoyl-8-^{13}C-adenosine (S3)

N^6-Benzoyl-8-^{13}C-adenine **S2** (1.46 g, 6.07 mmol) and 1-*O*-Acetyl-2,3,5-tri-*O*-benzoyl-β-*D*-ribofuranose (ATBR, 3.06 g, 6.07 mmol) were co-evaporated in anhydrous toluene. The residue was suspended in anhydrous toluene (30 mL) and *N*,*O*-bis(trimethylsilyl)acetamide (BSA, 3.7 g, 4.5 ml, 18.21 mmol) was added. The supension was stirred at 105°C under an argon atmosphere and turned in a clear solution after 30 min. Then, trimethylsily trifluoromethanesulfonate (TMSOTf, 3.89 ml, 21.3 mmol) was added and stirring was continued for another 30 min. The solvents were evaporated and after drying in high vacuum, the oily resiude was dissolved in methylene chloride. The organic phase was washed with saturated sodium bicarbonate solution, dried over anhydrous sodium sulfate, and then methylene chloride was evaporated. The resulting foam was purified by column chromatography (CC, silica, CH_2Cl_2/acetone 99/1 CH_2Cl_2/acetone 95/5).

<u>Yield:</u> 3.1 g (4.53 mmol, 75%)
<u>TLC:</u> 9/1 CH_2Cl_2/acetone, R_f = 0.5
<u>^{1}H-NMR (300 MHz, $CDCl_3$, 25°C)</u>
δ 8.71 (s, 1H, C(2)*H*); 8.17 (d, 1H, C(8)*H*, $^1J_{CH}$=213 Hz); 8.11-7.87 (m, 10H, C(arom)*H*); 7.63-7.34 (m, 10H, C(arom)*H*); 6.49 (m, 1H, C(1')*H*); 6.43 (m, 1H, C(2')*H*); 6.26 (m, 1H, C(3')*H*); 4.85 (m, 1H, C(4')*H*); 4.93 + 4.71 (dd, 2H, C(5')H_2) ppm.
<u>^{13}C-NMR (75 MHz, $CDCl_3$, 25°C)</u>
δ 153.47 (C(2)*H*); 141.98 (C(8)*H*); 130.27 (C(arom)*H*); 128.98 (C(arom)*H*) ppm.

N^6-Benzoyl-8-^{13}C-adenosine (S4)

Compound **S3** (1.76 g, 2.6 mmol) was dissolved in a mixture of pyridine and ethanol (30 ml, 1:1) and stirred in an ice bath at 0°C. A 2 M NaOH solution (25 ml in H_2O/EtOH 1/1) was added and the reaction was stirred for 20 min. The strongly akaline solution was neutralized using DOWEX 50WX8-100 H^+-form. The DOWEX resin was filtered off and washed with pyridine/ethanol 4:1 (200 ml). The solvents were evaporated and the crude yellow residue was co-evaporated twice with toluene. The slightly yellow residue was triturated with diethylether/CH_2Cl_2 1/1 (100 ml). the resulting precipitate was isolated by filtration and dried in high vacuum. The crude product was directly used in the next step.

Yield: 957 mg (2.6 mmol, 100%)

N^6-Benzoyl-5'-*O*-(4,4'-dimethoxytrityl)-(8-^{13}C)adenosine (S5)

The crude product **S3** (1.0 g, 2.69 mmol) from the previous step was co-evaporated three times with pyridine and dried in high vacuum for 30min. The oily residue was dissolved in anhydrous pyridine (10 ml) and stirred under argon at room temperature. Then, 4,4'-dimethoxytrityl chloride (DMT-Cl, 910 mg, 2.69 mmol) was added in three portions over a period of 1 h. After another 3 hours thin layer chromatography (TLC) showed complete conversion and the solvent was evaporated. The residue was co-evaporated three times with toluene. The resiude was dissolved in methylene chloride and the organic phase was washed once with saturated sodium bicarbonate solution and dried over anhydrous sodium sulfate. Compound **S5** was purified by CC using a gradient of methanol in CH_2Cl_2 from 1% to 7%.

Yield: 1.34 g (1.99 mmol, 74%)
TLC: 9/1 CH_2Cl_2/MeOH, R_f=0.5
^{1}H-NMR (300 MHz, $CDCl_3$, 25°C):
δ 9.09 (s, 1H, N*H*CO); 8.75 (s, 1H, C(2)*H*); 8.24 (d, 1H, C(8)*H*, $^1J_{CH}$=210 Hz); 7.72-7.48 (m, 4H, C(arom)*H*); 7.36-7.08 (m, 9H, C(arom)*H*); 6.90-6.70 (m, 5H, C(arom)*H*); 6.05 (m, 1H, C(1')*H*); 4.89 (m, 1H, C(2')*H*); 4.45 (m, 1H, C(3')*H*); 4.43 (m, 1H, C(4')*H*); 3.75 (s, 6H, C(arom)OCH_3); 3.44 + 3.29 (dd, 2H, C(5')H_2) ppm.
^{13}C-NMR (75 MHz, CDCl3, 25°C):
δ 141.55 (*C*(8)H) ppm.

N^6-Benzoyl-5'-*O*-(4,4'-dimethoxytrityl)-2'-*O*-[(triisopropylsilyl)oxy]methyl (8-^{13}C)adenosine (S6)

Compound **S5** (646 mg, 1.05 mmol) was dissolved in a mixture of 1,2-dichloroethane (DCE, 7 ml) and *N*,*N*-diisopropylethylamine DiPEA (610 µl, 3.68 mmol). Then, di-*tert.*-butyltin dichloride (352 mg, 1.2 mmol) was added and the reaction was stirred under argon at room temperature for 30 minutes. Triisopropylsilyloxymethyl chloride (TOM-Cl, 281 mg, 1.3 mmol) was added and the reaction was heated to 80°C for 40 min. The reaction was diluted with CH_2Cl_2 and washed once with saturated $NaHCO_3$ solution. The organic phase was dried over anhydrous Na_2SO_4. The crude **S6** was purified by column chromatography using a gradient of ethylacetate/hexane from 1/4 to 6/4. The 3'-regioisomer of **S6** was also collected and deprotected using 1M tetrabutylammonium fluoride (TBAF) in acetonitrile. This material was combined with unreacted **S5** and and again used for the tomylation reaction.

Yield: 440 mg (0.51 mmol, 49%, combined yield from 3 reactions)
TLC: 6/4 ethyl acetate/hexane, R_f = (2'-TOM)=0.6, R_f = (3'-TOM)=0.4
^{1}H-NMR (300 MHz, DMSO-d_6, 25°C):
δ 11.2 (s, 1H, N*H*CO); 8.59 (d, 1H, C(8)*H*, $^1J_{CH}$=214 Hz); 8.59 (s, 1H, C(2)*H*); 8.04 (d, 2H, C(arom)*H*); 7.67-7.51 (m, 3H, C(arom)*H*); 7.37-7.19 (m, 9H, C(arom)H); 6.85-6.80 (d, 4H, C(arom)H); 6.19 (d, 1H, C(1')*H*); 5.32 (d, 1H, C(3')O*H*); 5.10 (m, 1H, C(2')*H*); 4.98 + 4.93 (2d, 2H, OCH_2O, $^2J_{HH}$=5.1 Hz); 4.50 (m, 1H, C(3')*H*); 4.15 (m, 1H, C(4')*H*); 3.72 (s, 6H, C(arom)OCH_3); 3.28 + 3.22 (dd, 2H, C(5')H_2); 1.05, 0.86-0.82 (m, 21H, SiCH(CH_3)$_3$) ppm.

^{13}C-NMR (75 MHz, DMSO-d_6, 25°C):
δ 150.7 (*C*(2)H); 143.6 (*C*(8)H); 128.5, 128.2 127.9, 127.5, 126.5, 112.7 (*C*(arom)H); 87.4 (O*C*H_2O); 85.9 (*C*(1')H); 83.3 (*C*(4')H); 75.8 (*C*(2')H); 67.8 (*C*(3')H); 62.2 (*C*(5')H_2); 54.5 (C(arom)O*C*H_3); 16.6 (SiCH(*C*H_3)$_3$); 10.5 (Si*C*H(CH_3)$_3$) ppm.

N^6-Benzoyl-5'-*O*-(4,4'-dimethoxytrityl)-2'-*O*-[(triisopropylsilyl)oxy]methyl (8-^{13}C)-adenosine 3'-*O*-(2-cyanoethyl-*N,N*-diisopropylphosphoramidite) (S7)

S6 (440 mg, 0.51 mmol) was dissolved in a mixture of anhydrous CH_2Cl2 (4 ml) and DiPEA (348 µl, 2.1 mmol). The solution was stirred for 5 minutes at room temperature under argon. Then**,** 2-cyanoethyl *N,N*-diisopropylchlorophosphoramidite (CEP-Cl, 147 mg, 0.62 mmol) was added and the solution was stirred 4 h, before a small amount of methanol was added to stop the reaction. After dilution with methylene chlorid, the organic phase was washed with saturated sodium bicarbonate solution, and then dried over anhydrous sodium sulfate. The organic solvent was evaporated and the crude prodcut was purified by column chromatography using a gradient ethyl acetate/hexane from 1/4 to 6/4 (+ 2% NEt_3).

Yield: 389 mg (0.37 mmol, 72%, white foam)
TLC: 1/1 hexane/ethyl acetate, R_f = 0.5
^{1}H-NMR (300 MHz, $CDCl_3$, 25°C):
δ 8.94 (s, 2H, N*H*CO); 8.71, 8.68 (2s, 2H, C(2)*H*); 8.19, 8.16 (2d, 2H, C(8)*H*, $^1J_{CH}$=212 Hz); 8.02 (d, 4H, C(arom)*H*); 7.63-7.50 (m, 6H, C(arom)*H*); 7.43-7.18 (m, 18H, C(arom)*H*); 6.81-6.76 (m, 8H, C(arom)*H*); 6.21 (m, 2H, C(1')*H*); 5.23 (m, 2H, C(2')*H*); 4.98 + 4.93 (2d, 4H, OC*H*$_2$O, $^2J_{HH}$=5.05 Hz); 4.72 (m, 2H, C(3')*H*); 4.43, 4.38 (m, 2H, C(4')*H*); 3.78, 3.77 (2s, 12H, C(arom)OC*H*$_3$); 4.01-3.82 (m, 2H, POC*H*$_2$); 3.71-3.51 (m, 8H, C(5')*H*, POC*H*$_2$, NC*H*(C*H*$_3$)$_2$); 3.38-3.32 (m, 2H, C(5')*H*); 2.65 (m, 2H, 1H of CH_2CN, $^2{}_{JHH}$=6.4); 2.38 (m, 2H, CH_2CN, $^2J_{HH}$=6.4); 1.22, 1.20, 1.18, 1.10, 1.08 (3d, 24H, NCH(C*H*$_3$)$_2$; $^3J_{HH}$=6.4); 0.91, 0.90 (2s, 42H, SiCH(C*H*$_3$)$_2$) ppm.
^{13}C-NMR (75 MHz, $CDCl_3$, 25°C):
δ 152.8 (*C*(2)H); 142.6, 142.5 (*C*(8)H); 134.6, 130.1, 128.9, 128.4, 128.0, 127.7 (*C*(arom)H); 113.7 (*C*(arom)H); 89.4 (O*C*H_2O); 88.0 (*C*(1')H); 84.1 (*C*(4')H); 77.7 (*C*(2')H); 72.2 (*C*(3')H); 63.9 (*C*(5')H); 58.5 (PO*C*H_2); 55.4 (C(arom)O*C*H_3); 42.9 (N*C*H(CH_3)$_2$); 24.9 (NCH(*C*H_3)$_2$); 20.7 (*C*H_2CN); 18.1 (SiCH(*C*H_3)$_2$); 12.3 (Si*C*H(CH_3)$_2$) ppm.
^{31}P-NMR (121 MHz, $CDCl_3$, 25°C):
δ 151.57, 150.78 ppm.
ESI-MS:
1061.3 $[M + H]^+$, 1083.5 $[M + Na]^+$

Synthesis of N^2-iso-butyryl-8-^{13}C-guanine (S9)

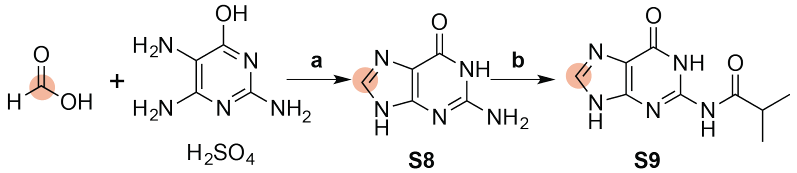

Supporting Scheme 3. Synthesis of N^2-*iso*-butyryl-8-^{13}C-guanine **S9**. Reaction conditions: **a** morpholine, 3 h, 100-200 °C, 94%. **b** isobutyric anhydride (iBu$_2$O) in *N*,*N*-dimethylacetamide (DMA), 4 h, 150 °C, 77%. Orange cirlce = ^{13}C.

8-^{13}C-Guanine (S8)

Morpholine (1.8 g, 20.7 mmol) and ^{13}C-formic acid (798 µl, 20.7 mmol) were combined at 0°C. The clear solution turned yellow and crystallized after a few minutes. 2,4,5-Triamino-6-hydroxypyrimidine sulfate (1.65 g, 6.9 mmol) was added the mixture heated to 100°C. After 40 min. the temperature was raised to 200°C. The reaction was continued for 2 h. After cooling to room temperature ethanol was added and the mixture heated to 100°C for 30 min. The precipitate was isolated by filtration and transferred into a round bottom flask. The crude guanine was suspended in water and 2 ml concentrated aqueous ammonia (28%) was added. The suspension was concentrated on a rotary evaporator until a pH 7 was reached and then cooled to 4°C overnight. 8-^{13}C-Guanine **S8** was isolated by filtration and then dried in high vacuum.

<u>Yield:</u> 983 mg (6.5 mmol, 94%)

<u>^{1}H-NMR (300 MHz, DMSO-d$_6$, 25°C):</u>
δ 8.91 (d, 1H, C(8)*H*, $^1J_{CH}$=219 Hz) ppm.
<u>^{13}C-NMR (75 MHz, DMSO-d$_6$, 25°C):</u>
δ 137.54 (*C*(8)H) ppm.

***N*^{2}-Isobutyryl-(8-^{13}C)-guanine (S9)**

S8 (1.77 g, 11.63 mmol) was suspended in *N*,*N*-dimethylacetamide (DMA, 15 ml). To the suspension isobutyric anhydride (iBu$_2$O, 6.2 ml, 37.2 mmol) was added and the mixture was refluxed at 170°C under argon. After three hours, the solution was cooled to room temperature and the solvent was removed by distillation. The residual oil was co-evaporated twice with methanol and recrystallized in ethanol/H_2O (1/1). After filtration **S9** was dried under high vacuum.

<u>Yield:</u> 2.0 g (9.0 mmol, 77%)

<u>^{1}H-NMR (300 MHz, DMSO-d$_6$, 25°C):</u>
δ 13.33 (br. s, 1H, N*H*); 12.03 (s, 1H, N*H*); 11.49 (s, 1H, N*H*); 7.99 (d, 1H, C(8)*H*, $^1J_{CH}$=211 Hz); 2.72 (sept, 1H, C*H*(CH$_3$)$_2$); 1.09 (d, 6H, CH(C*H*$_3$)$_2$) ppm.

<u>^{13}C-NMR (75 MHz, DMSO-d$_6$, 25°C):</u>
δ 137.7 (*C*(8)H) ppm.

Synthesis of N²-iso-butyryl-5'-O-DMT-2'-O-TOM-8-¹³C-guanosine 3'-O-CEP (S14)

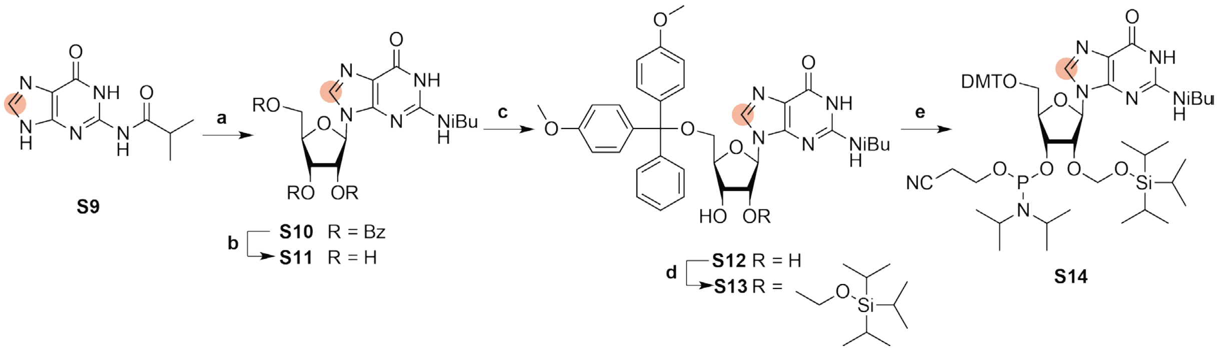

Supporting Scheme 4. Synthesis of N²-isobutyryl-5'-*O*-DMT-2'-*O*-TOM-8-¹³C-guanosine 3'-*O*-CEP **S14**. Reaction conditions: **a** 1-*O*-Acetyl-2,3,5-tri-*O*-benzoyl-β-*D*-ribofuranose (ATBR), *N*,*O*-Bis(trimethylsilyl)acetamide (BSA), trimethylsily trifluoromethanesulfonate (TMSOTf) in toluene, 1 h, 105°C, 83%. **b** 1N NaOH in pyridine/ethanol 1/1, 30 min, 0°C, 99%. **c** 4,4'-Dimethoxytrityl chloride (DMT-Cl) in pyridine, 4 h, rt, 70%. **d** Di-tert.-butyltin dichloride, *N.N*-diisopropylethylamine (DiPEA), triisopropylsilyloxymethyl chloride (TOM-Cl), 1 h, 80°C, 52%. **e** 2-Cyanoethyl *N,N*-diisopropylchlorophosphoramidite (CEP-Cl), DiPEA in methylene chloride, 16 h, rt, 77%. Orange cirlce = ¹³C.

***N*²-Isobutyryl-2',3',5'-tri-*O*-benzoyl-(8-¹³C)-guanosine (S10)**

Compound **S9** (1.0 g, 4.5 mmol) and ATBR (2.27 g, 4.5 mmol) were suspended in anhydrous toluene (20 ml). BSA (6.62 ml, 27 mmol) was added and the reaction was heated to 105°C under argon. After 1h the solution was allowed to cool to room temperature and (TMS)OTf (2.46 ml, 13.5 mmol) was added. The solution was heated again to 105°C and stirred for 1 h. The solution was diluted with CH_2Cl_2 and washed with saturated $NaHCO_3$ solution. Dried over anhydrous Na_2SO_4, the solvents were evaporated and the crude product was purified by column chromatography using a gradient of MeOH in CH_2Cl_2 from 0% to 3%.

Yield: 2.5 g (3.75 mmol, 83%)
TLC: 9/1 CH_2Cl_2/MeOH, R_f = 0.6
¹H-NMR (300 MHz, $CDCl_3$, 25°C):
δ 11.90 (s, 1H, N*H*); 9.11 (s, 1H, N*H*CO); 7.78 (d, 1H, C(8)*H*, $^1J_{CH}$=213 Hz); 8.00-7.81 (m, 8H, C(arom)*H*); 7.60-7.30 (m, 7H, C(arom)*H*); 6.57 (m, 1H, C(3')*H*); 6.36 (m, 1H, C(2')*H*); 6.18 (m, 1H, C(1')*H*); 4.86 (m, 1H, C(4')*H*); 4.95 + 4.79 (dd, 2H, C(5')*H*₂); 2.71 (sept, 1H, C*H*(CH_3)₂); 1.31 (d, 6H, CH(C*H*₃)₂) ppm.
¹³C-NMR (75 MHz, $CDCl_3$, 25°C):
δ 138.89 (*C*(8)H); 134.2 (*C*(arom)H); 129.6 (*C*(arom)H); 128.1 (*C*(arom)H); 88.0 (*C*(1')H); 74.0 *(C*(2')H); 71.2 (*C*(3')H) 69.6 (*C*(4')H); 62.4 (*C*(5')H₂); 36.46 (*C*H(CH_3)₂); 18.85 (CH(*C*H₃)₂) ppm.

***N*²-Isobutyryl-(8-¹³C)-guanosine (S11)**

Sodium ethoxide (NaOEt, 2.4 g, 35.3 mmol) was dissolved in 34 ml pyridine/EtOH 2/3 and **S10** (2.8 g, 4.2 mmol), dissolved in 20 ml pyridine/EtOH 2/3, was added. The reaction was stirred for 25 minutes at room temperature. The strongly alkaline solution was neutralized by DOWEX 50WX8-100 H^+-form. The DOWEX was removed by filtration and washed with pyridine/ethanol 3/2. The solvents were evaporated and co-evaporated twice with toluene. The residue was dissolved in water and the aqueous phase was washed with CH_2Cl_2. The aqeous phase was evaporated and the oily residue was co-evaporated with methanol. The resulting orange foam was dried under high vacuum. The crude product was directly used in the next step.

Yield: 1.47 g (4.15 mmol, 99%)

N^2-Isobutyryl-5'-*O*-(4,4'-dimethoxytrityl)-(8-^{13}C)-guanosine (S12)

S11 (870 mg, 2.46 mmol) was co-evaporated three times with pyridine before being suspended in anhydrous pyridine (15 ml). The reaction was stirred under argon at room temperature for ten minutes. Then, DMT-Cl (1.25 g, 3.68 mmol) was added in two portions while the progress of the reaction was monitored with TLC. After stirred 4 h the solvent was evaporated and the residue was co-evaporated three times with toluene and then dried in high vacuum. The oily residue was then dissolved in methylene chloride and the organic phase was washed with saturated sodium bicarbonate, dried over anhydrous sodium sulfate. After evaporation crude **S12** was purified by column chromatography using a gradient from 0% to 10% of methanol in CH_2Cl_2.

Yield: 1.20 g (1.86 mmol, 75%)
TLC: 9/1 CH_2Cl_2/MeOH, R_f=0.4
^{1}H-NMR (300 MHz, $CDCl_3$, 25°C):
δ 7.76 (d, 1H, C(8)*H*, $^1J_{CH}$=214 Hz); 7.45-7.12 (m, 9H, C(arom)*H*); 6.80-6.75 (m, 4H, C(arom)*H*); 5.83 (m, 1H, C(1')*H*); 5.14 (m, 1H, C(2')*H*); 4.52 (m, 1H, C(3')*H*); 4.28 (m, 1H, C(4')*H*); 3.74 (s, 6H, C(arom)OCH_3); 3.45 + 3.14 (dd, 2H, C(5')H_2); 2.05 (sept, 1H, C*H*(CH_3)$_2$); 0.94 + 0.78 (2d, 6H, CH(CH_3)$_2$) ppm.
^{13}C-NMR (75 MHz, $CDCl_3$, 25°C):
δ 139.1 (*C*(8)H); 137.65 (*C*(arom)H); 130.1 (*C*(arom)H); 128.3 (*C*(arom)H); 127.9 (*C*(arom)H); 113.3 (*C*(arom)H); 90.1 (*C*(1')H); 85.4 (*C*(4')H); 73.9 (*C*(2')H) 71.8 (*C*(3')H); 63.6 (*C*(5')H); 55.4 (C(arom)O*C*H_3); 36.1 (*C*H(CH_3)$_2$); 18.7 (CH(*C*H_3)$_2$) ppm.

***N^2*-Isobutyryl-5'-*O*-(4,4'-dimethoxytrityl)-2'-*O*-[(triisopropylsilyl)oxy]methyl-(8-^{13}C)-guanosine (S13)**

S12 (1.84 g, 2.80 mmol) was dissolved in a mixture of 1,2-DCE (22 ml) and DiPEA (1.77 ml, 10.7 mmol). Then, di-*tert*.-butyltin dichloride (893 mg, 2.94 mmol) was added and the reaction was allowed to stir under argon at room temperature for 30 minutes. TOM-Cl (712 mg, 3.22 mmol) was added and the reaction was heated to 80°C for 40 minutes and stirred under argon. The reaction was diluted with CH_2Cl_2 and washed once with saturated sodium bicarbonate solution. The organic phase was dried over anhydrous Na_2SO_4, filtered, and evaporated. The crude **S13** was purified by column chromatography using a gradient of ethyl acetate/hexane from 3/7 to 7/3.

Yield: 1.22 g (1.45 mmol, 52%)
TLC: 7/3 ethyl acetate/hexane, R_f=0.6
^{1}H-NMR (300 MHz, $CDCl_3$, 25°C):
δ 11.91 (s, 1H, N*H*); 7.78 (d, 1H, C(8)*H*, $^1J_{CH}$=211 Hz); 7.42-7.21 (m, 9H, C(arom)*H*); 6.82-6.77 (m, 4H, C(arom)*H*); 5.89 (m, 1H, C(1')*H*); 5.14 + 4.95 (dd, 2H, OCH_2O, $^1J_{HH}$=4.75 Hz); 5.10 (m, 1H, C(2')*H*); 4.57 (m, 1H, C(3')*H*); 4.23 (m, 1H, C(4')*H*); 3.76 (2s, 6H, C(arom)OCH_3); 3.55 + 3.10 (dd, 1H, C(5')H_2, $^1J_{HH}$=2.8 Hz); 2.98 (m, 1H, C(3')O*H*); 1.44 (sept, 1H, C*H*(CH_3)$_2$); 1.21-0.98 (m, 21H, SiCH(CH_3)$_2$); 0.87 + 0.65 (2d, 6H, CH(CH_3)$_2$) ppm.
^{13}C-NMR (75 MHz, $CDCl_3$, 25°C):
δ 139.39 (*C*(8)H); 130.3 (*C*(arom)H); 128.3 (*C*(arom)H); 127.5 (*C*(arom)H); 113.5 (*C*(arom)H); 91.6 (O*C*H_2O); 86.7 (*C*(1')H); 84.6 (*C*(4')H); 81.5 (*C*(2')H) 71.2 (*C*(3')H); 64.0 (*C*(5')H_2); 55.4 (C(arom)O*C*H_3); 36.7 *C*H(CH_3)$_2$); 18.7 (CH(*C*H_3)$_2$); 18.05 (SiCH(*C*H_3)$_2$); 12.02 (Si*C*H(CH_3)$_2$) ppm.

N^2-Isobutyryl-5'-*O*-(4,4'-dimethoxytrityl)-2'-*O*-[(triisopropylsilyl)oxy]methyl-(8-^{13}C)-guanosine 3'-*O*-(2-cyanoethyl-*N*,*N*-diisopropylphosphoramidite) (S14)

S14 (2.0 g, 2.37 mmol) was dissolved in a mixture of anhydrous CH_2Cl_2 (10 ml) and *N*,*N*-dimethylethylamine (DMEA, 1.28 ml, 11.9 mmol). Then, CEP-Cl (1.12 g, 4.74 mmol) was added to the solution and stirring continued for 16 h hours under argon. The reaction was quenched with a few drops of methanol. Crude **S14** was purified by column chromatography using a gradient of ethyl acetate/hexane from 1/1 to 8/2 (+1% NEt_3) and then dried in high vacuum.

Yield: 1.9 g (1.82 mmol, 77%)
TLC: 7/3 ethyl acetate/hexane, R_f = 0.8
^{1}H-NMR (300 MHz, $CDCl_3$, 25°C):
δ 8.23 (s, 2H, N*H*); 7.83 (s, 2H, N*H*CO); 7.84,7.79 (2d, 2H, C(8)*H*, $^1J_{CH}$=214 Hz); 7.55-7.45 (m, 4H, C(arom)*H*); 7.42-7.36 (m, 12H, C(arom)*H*); 6.81-6.76 (m, 10H, C(arom)*H*); 5.96, 5.84 (m, 2H, C(1')*H*); 5.16, 5.07 (m, 1H, C(2')*H*); 4.98 + 4.90 (dd, 4H, OC*H*$_2$O, $^1J_{HH}$=5.3 Hz); 4.60, 4.54 (m, 2H, C(3')*H*); 4.33, 4.23 (m, 2H, C(4')*H*); 4.02, 3.92 (m, 2H, POC*H*$_2$); 3.77, 3.76 (2s, 12H, C(arom)OC*H*$_3$); 3.66-3.46 (m, 8H, NC*H*(CH_3)$_2$, C(5'')*H*, POC*H*$_2$), 3.28 (m, 2H, C(5')*H*); 2.74 (td, 2H, NCC*H*$_2$, $^1J_{HH}$=7.1 Hz); 2.26 (t, 2H, NCC*H*$_2$, $^1J_{HH}$=6.3 Hz); 1.89, 1.69 (sept, 2H, C*H*(CH_3)$_2$); 1.30-1.00 (m, 24H, NCH(C*H*$_3$)$_2$); 0.96-0.90 (m, 42H, SiCH(C*H*$_3$)$_2$); 1.00, 0.77, 0.75 (2d, 12H, CH(C*H*$_3$)$_2$) ppm.
^{13}C-NMR (75 MHz, $CDCl_3$, 25°C):
δ 178.57 (NH*C*O); 158.07 (*C*(arom)); 145.21 (*C*(arom)); 138.08 (C(8)H); 130.26 (*C*(arom)H); 128.44 (*C*(arom)H); 128.17 (C(arom)H); 127.26 (*C*(arom)H); 113.35 (*C*(arom)H); 89.59 (O*C*H$_2$O); 86.41 (*C*(1')H); 84.61 (*C*(4')H); 76.73 (*C*(2')H) 71.44 (*C*(3')H); 63.75 (*C*(5')H$_2$); 57.48 + 59.17 (PO*C*H$_2$); 55.53 (C(arom)O*C*H$_3$); 43.59 (NC*H*(CH_3)$_2$); 36.32 (C*H*(CH_3)$_2$); 24.94 (NCH(CH*3*)$_2$); 20.48 (NC*C*H$_2$); 18.83 (CH(C*H*$_3$)$_2$); 17.95 (Si*C*H(CH_3)$_2$); 12.21 (SiCH(*C*H$_3$)$_2$) ppm.
^{31}P-NMR (121 MHz, $CDCl_3$, 25°C):
δ 150.62, 150.36 ppm.
ESI-MS:
1043.3 $[M + H]^+$, 1065.5 $[M + Na]^+$

Synthesis of 6-^{13}C-5D-pyrimidine RNA phosphoramidites

Synthesis of 5D-6-^{13}C-uracil (S18)

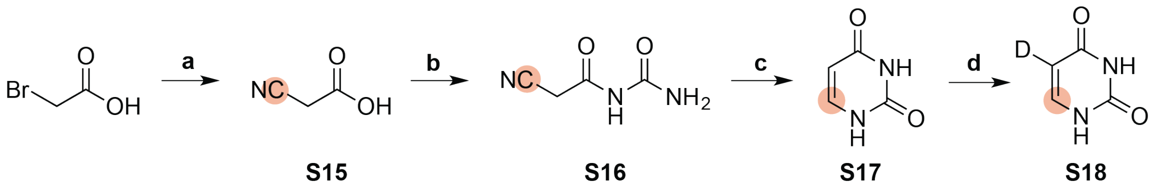

Supporting Scheme 5. Synthesis of 6-^{13}C-5D-uracil **S18**. Reaction conditions: **a** Na_2CO_3, $K^{13}CN$ in H_2O, 16 h, 80°C to rt, 96%. **b** urea in acetic anhydride, 90 %, 30 min, 93%. **c** Pd/$BaSO_4$ in 50 % aqueous acetic acid, rt, 16 h, 71%. **d** triethylamine in D_2O, 90 h, 110°C, 100%. Orange cirlce = ^{13}C.

Steps **a** to **c** were previously published. For experimental details please refer to reference [1].

5D-6-^{13}C-uracil (S18)

6-^{13}C-uracil **S17** (1.47 g, 13.06 mmol) was dissolvde in a mixture of D_2O (60 ml) and triethylamine (9 ml) and refluxed for 90 h at 110°C under argon. ^{1}H-NMR showed complete disappearance of the C5 proton. All solvents were evaporated and **S18** was dried in high vacuum.

Yield: 1.49 g (13.06 mmol, 100%)
^{1}H-NMR (300 MHz, DMSO-d_6, 25°C):
δ 10.64 (s, 2H, N*H*); 7.39 (m, 1H, C(6)*H*, $^1J_{CH}$=180 Hz) ppm.
^{13}C-NMR (75 MHz, $CDCl_3$, 25°C):
δ 164.3 (*C*(4)); 151.5 (*C*(2)); 142.2 (*C*(6)H); 100.2 (m, *C*(5)D) ppm.

Synthesis of 5'-O-DMT-2'-O-TOM-6-^{13}C-5D-uridine 3'-O-CEP (S23)

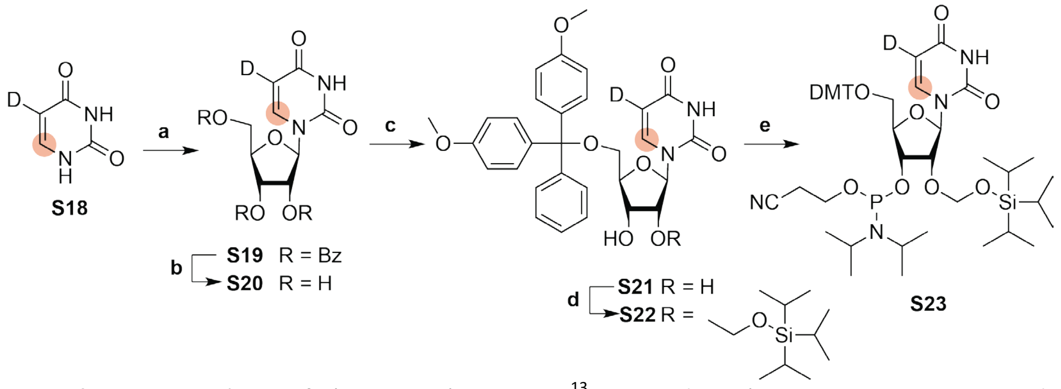

Supporting Scheme 6. Synthesis of 5'-*O*-DMT-2'-*O*-TOM-6-^{13}C-5D-uridine 3'-*O*-CEP **S23**. Reaction conditions: **a** ATBR, BSA, TMSOTf in acetonitrile, 1 h, 60°C, 98%. **b** methylamine in ethanol, 16 h, rt, 100%. **c** DMT-Cl in pyridine, 4 h, rt, 70%. **d** TOM-Cl, DiPEA, di-*tert.*-butyltin dichloride in 1,2-DCE, 1 h, 80°C, 40%. **e** CEP-Cl, DiPEA in methylene chloride, 2 h, rt, 80%.Orange cirlce = ^{13}C.

All steps were previously published for the 6-^{13}C-derivative. For experimental details please refer to reference [1].

Synthesis of N⁴-Acetyl-5'-O-DMT-2'-O-TOM-6-¹³C-5D-cytidine 3'-O-CEP (S27)

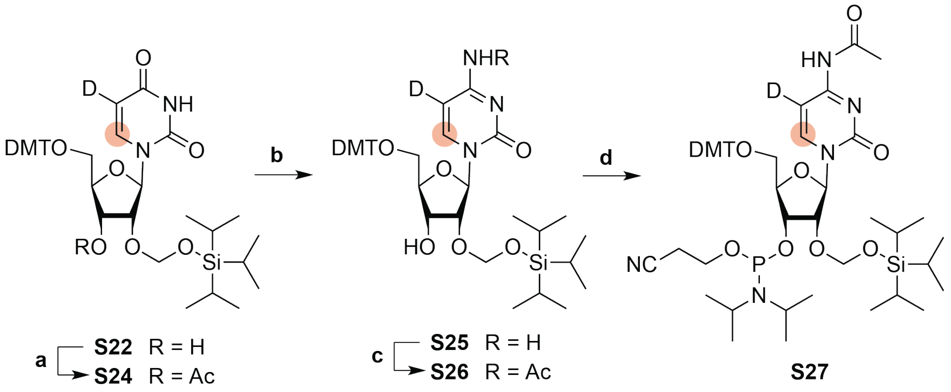

Supporting Scheme 7. Synthesis of N^4-Acetyl-5'-*O*-DMT-2'-*O*-TOM-6-^{13}C-5D-cytidine 3'-*O*-CEP **S27**. Reaction conditions: **a** acetic anhydride in pyridine, 3 h, rt, 90%. **b** 2,4,6-Triisopropylbenzenesulfonyl chloride, triethylamine in methylene chloride, 2 h, rt, then, aqueous ammonia (28%) in THF, 18 h, rt, the nmethylamine in ethanol, 1 h, rt, 68%. **c** acetic anhydride in DMF, 22 h, rt, 90%. **d** CEP-Cl, DiPEA in methylene chloride, 2 h, rt, 90%. Orange cirlce = ^{13}C.

All steps were previously published for the 6-^{13}C-derivative. For experimental details please refer to reference [1].

Solid phase synthesis of stable isotope modified RNA and DNA

Solid phase synthesis

The $^{13}C/^{2}H$-modified phosphoramidites were used in combination with standard 2′-*O*-TOM protected RNA or standard DNA building blocks (ChemGenes) to synthesize RNA sequence 1**a** and DNA sequence **2a**. Custom primer support PS 200 (GE Healthcare) with an average loading of 80 µmol g^{-1} were used. The sequences were synthesized on an ABI 391 PCR Mate using self-written RNA/DNA synthesis cycles (available on request). Amidite (0.1 M) and activator (5-benzylthio-1*H*-tetrazole, 0.25 M) solutions were dried over freshly activated molecular sieves overnight.

DNA deprotection

The removal of protecting groups and the cleavage from solid support was achieved by treatment with aqueous methylamine (40%, 650 µL) and aqueous ammonia (28%, 650 µL) at 310 K for 1.5 h. After evaporation of the alkaline deprotection solution crude DNA was dissolved in 1 mL water.

RNA deprotection

The removal of protecting groups and the cleavage from solid support was achieved by treatment with aqueous methylamine (40%, 650 µL) and ethanolic methylamine (8 M, 650 µL) at room temperature for 6 hours. After evaporation of the alkaline deprotection solution the 2′-*O*-protecting groups were removed by adding 1 M TBAF (tetrabutylammonium fluoride) in THF (1200 µL). After 16 h at 310 K the reaction was quenched by the addition of 1 M triethylammonium acetate (TEAA, pH 7.0, 1200 µL). The volume was reduced to approximately 1 mL and then applied on a HiPrep 26/10 desalting column (GE Healthcare). The crude RNAs were eluted with water, evaporated to dryness and dissolved in 1 mL water.

Purification and quality check

The quality of the crude RNA and DNA was checked via anion exchange chromatography on a Dionex DNAPac PA-100 column (4x250 mm) using our standard eluents (Eluent A: 25 mM Tris.HCl, 6 M urea, pH 8.0; Eluent B: 25 mM Tris.HCl, 500 mM sodium perchlorate, 6 M urea, pH 8.0) and at elevated temperature (80 °C). Purification of the RNA/DNA sequences was achieved by applying the crude nucleic acid on a semi-preparative Dionex DNAPac PA-100 column (9x250 mm). The fractions containing the desired RNA or DNA were pooled and loaded on a C18 SepPak catridge (Waters) to remove HPLC buffer salts. The RNA sodium salt form was then eluted from the C18 column with water/acetonitrile (1/1, v/v) and lyophilized. The integrity of the RNAs was further checked by mass spectrometry on a Finnigan LCQ Advantage MAX ion trap instrumentation connected to an Amersham Ettan micro LC (GE Healthcare).

NMR spectroscopy

The RNA and DNA samples were lyophilized as the sodium salts and dissolved in 420 µL buffer (15 mM sodium phosphate, 25 mM NaCl, pH 6.5) in 90%/10% H_2O/D_2O. NMR experiments and ^{1}H-CPMG relaxation dispersion experiments were recorded either on an Agilent DD2 instruments operating at 500 MHz (11.7 T), on a Bruker 600 MHz (14.1 T) Avance II+ instrument equipped with a Prodigy cryo probe or on a 800 MHz (18.8 T) Bruker Advance III HD instrument equipped with a TCI cryo probe. For the A-site RNA **1** ^{1}H CPMG frequencies (ν_{CPMG}) of 100, 200, 200, 300, 400, 500, 500, 600, 700, 900, 1000, 1100, 1200, 1300, 1400, 1500, 1600, 1700, 1800, 1900, 1900 and 2000 Hz were used at 500 and 600 MHz and 100, 200, 200, 300, 400, 500, 500, 600, 700, 900, 1000, 1100, 1200, 1300, and 1400 at 800 MHz. The constant time relaxation delay T_{relax} was 20 ms for all experiments. 2048*72 (2048*64) complex data points were recorded at 500 MHz and 600 MHz (800 MHz) with a spectral width of 10 ppm in the carbon dimensions. The number of transients was 72 (64), and the interscan delay was set to 1.2 s (2.0 s) at 500 MHz and 600 MHz (800 MHz), respectively, yielding total experimental times between 30 and 50 hours. For the cTAR DNA **2** proton CPMG field strengths of 67, 133, 133, 200, 267, 333, 400, 467, 467, 533, 600, 667, 733, 800, 867, 933, 1000, 1066, 1133, 1133,and 1200 Hz were applied. The relaxation delay T_{relax} was set to 30 ms. 2048*80 complex data points were recorded with the spectral widths in the proton and carbon dimensions set to 10 ppm and 10 ppm, respectively. The number of transients was set to 72, and the interscan delay was set to 1.2 s yielding total experimental times of approx. 30 h.

Analysis of proton relaxation dispersion data

Spectral processing was performed using *nmrPipe* and the *nmrDraw* software package. Peak integration was performed using either the *nmrDraw* or *FuDA* software packages.[2-4]

Analysis of CPMG Relaxation dispersion data

For the A-site RNA the peak intentities as a function of CPMG frequencies, $I(\nu_{CPMG})$, were obtained using the FuDA software, whereas peak intensities for cTAR DNA were obtained by summing over 3x3 (t_1 x t_2) data points using the *serT*-script implemented in the *nmrPipe* package. Peak intensities were converted into effective relaxation rates via

$$R_{2,\mathrm{eff}}(\nu_{CPMG}) = -1/T_{\mathrm{relax}} \cdot \ln(I(\nu_{CPMG})/I_0)$$

where I_0 is the peak intensity in a reference spectrum recorded without the relaxation delay T_{relax}. The uncertainty of the $R_{2,\mathrm{eff}}(\nu_{CPMG})$ rates was propagated from the corresponding uncertainty of $I(\nu_{CPMG})$ and I_0 or set to 2% of $R_{2,\mathrm{eff}}(\nu_{CPMG})$, whichever was the largest.

Values of the exchange rate, k_{ex}, the population of the excited state, p_b, chemical shift differences between states, $\Delta\varpi$, and intrinsic relaxation rates $R^0_{2,\mathrm{eff}}$ that were assumed to be identical in the two exchanging sites, were extracted using the in-house written software *CATIA* by minimization of the following χ^2 target function:[5, 6]

$$\chi^2(\zeta) = \sum \frac{\left(\mathrm{R}^{\mathrm{calc}}_{2,\mathrm{eff}}(\zeta) - \mathrm{R}^{\mathrm{exp}}_{2,\mathrm{eff}}\right)^2}{\left(\sigma \mathrm{R}^{\mathrm{exp}}_{2,\mathrm{eff}}\right)^2}$$

where $R^{\mathrm{exp}}_{2,\mathrm{eff}}$ and $\sigma R^{\mathrm{exp}}_{2,\mathrm{eff}}$ are experimental effective relaxation rates and their uncertainties, respectively, $R^{\mathrm{calc}}_{2,\mathrm{eff}}(\zeta)$ are model relaxation rates obtained by numerical integration of the Bloch-McConnell equations over the relevant part of the pulse sequence for a two-site chemical exchange model, ζ denotes the set of adjustable model parameters and the summation is over the number of experimental data points.[7] In all fits of experimental dispersion profiles we have assumed that the intrinsic relaxation rates were the same for both exchanging states.

The exchange parameters k_{ex} and p_b were determined by only including those residues where (1) the two-site exchange model generated statistically significant improvements in fits over a model of no exchange at the 98% confidence level and (2) the maximum exchange contribution, $R_{2,\mathrm{eff}}$(100 Hz) - $R_{2,\mathrm{eff}}$(2000 Hz) > 3 s^{-1}. Thus, the relaxation dispersions of C7, C9, A10, A93, G94, U95 were included for the A-site RNA **1** and residues G4, A5, C19, G20, A21, C22 and C23 included for the cTAR DNA **2** for the determination of k_{ex} and p_b. In a subsequent fit, the obtained k_{ex} and p_b were kept fixed and the $\Delta\varpi$ for all residues determined. The uncertainties in the derived model parameters were obtained from the covariance matrix method.[8]

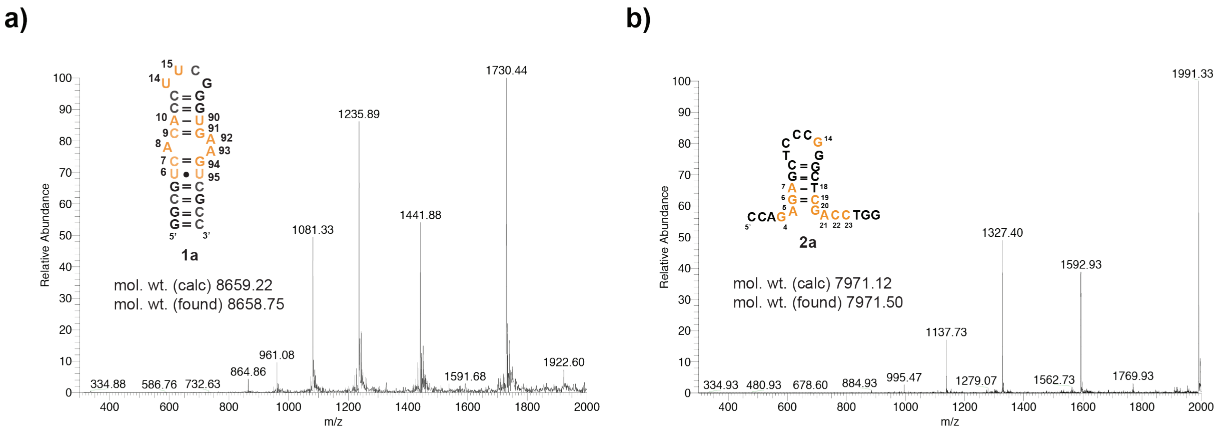

Supporting Figure 1. Mass spectrometric analysis of **a)** A-site RNA **1** and **b)** cTAR DNA **2**.

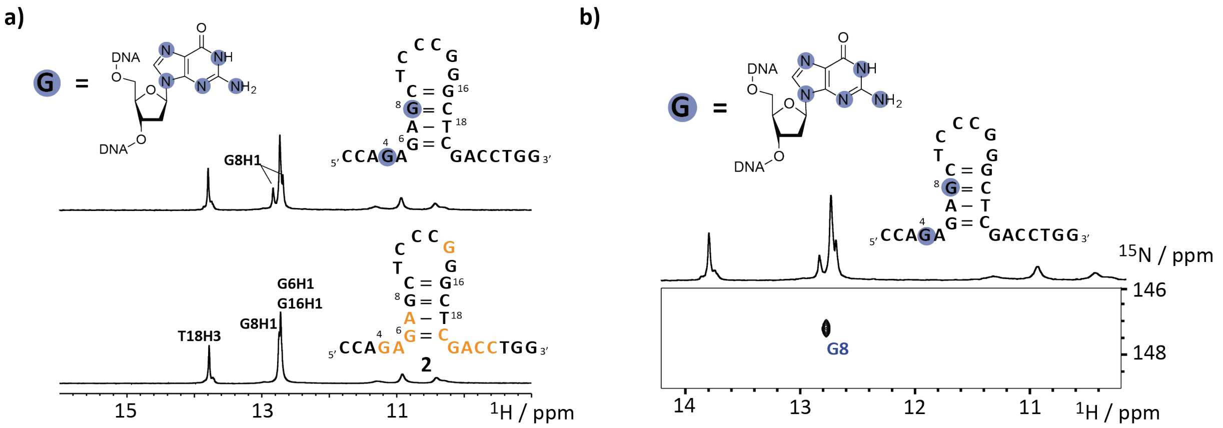

Supporting Figure 2. Assignment of imino proton resonances of cTAR DNA **2**. **a)** Imino proton spectrum of cTAR DNA. *Upper trace:* Guanosine 8 and 4 are selectively ^{15}N labeled. No ^{15}N splitted signal for residue G4 could be observed. *Lower trace:* Imino proton spectrum of $^{13}C/^{2}H$-labeled cTAR DNA **2**. Assignments were previously reported. **b)** ^{1}H-^{15}N-HSQC spectrum of the G4/G8 selectively ^{15}N labeled cTAR DNA. Only for residue G8 a crosspeak was observed in line with the partially melted form being the major species.

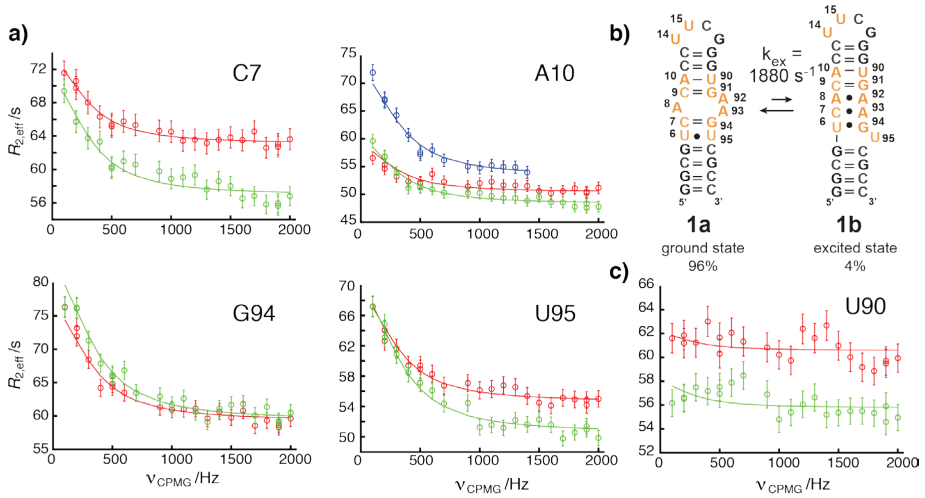

Supporting Figure 3. b) Selected proton relaxation dispersion curves of A-site RNA **1** at two/three magnetic field strengths (500 (red), 600 (green) and 800 (blue) MHz). Open circles represent data points, vertical bars the associated uncertainty. The fit is shown as a full-drawn line. **b)** Proposed exchange process for A-site RNA **1** with rates and populations derived from the relaxation dispersion curves in **a**. **c)** The relaxation dispersion profile of U90 exemplifies a "flat dispersion profile" when $\Delta\varpi \approx 0$ ppm, thereby showing the absent of homenuclear couplings and supporting the efficiency of the labelling scheme.

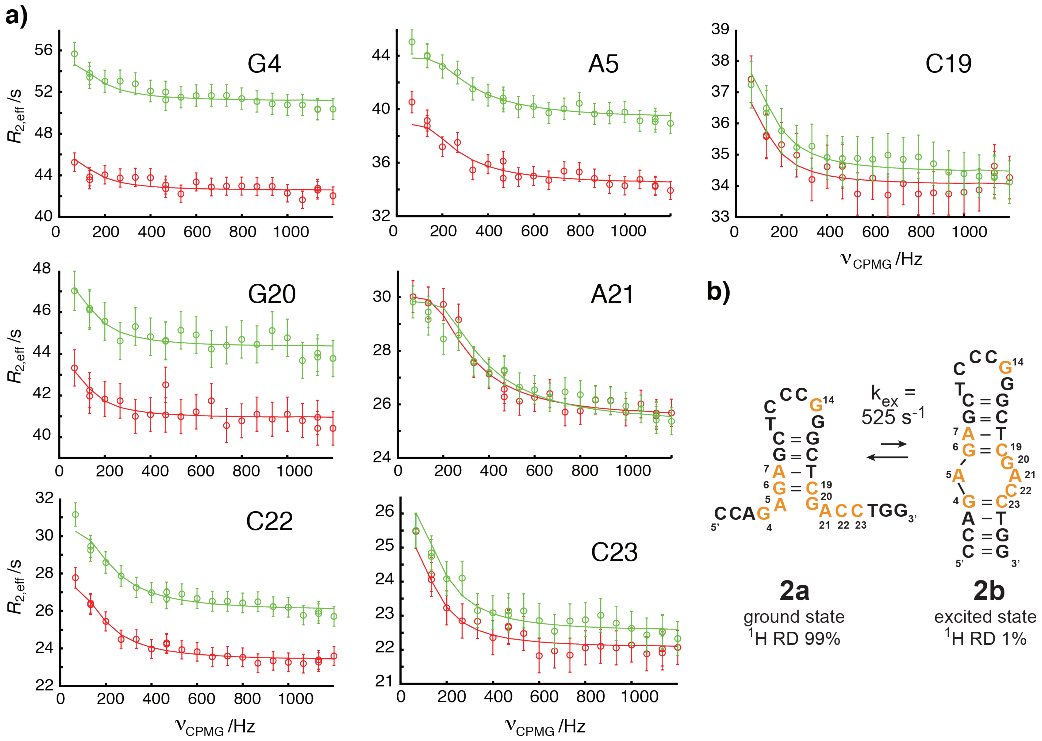

Supporting Figure 4. a) Proton relaxation dispersion curves of cTAR DNA **2** at two magnetic field strengths (500 (red) and 600 (green) MHz). Open circles represent data points, vertical bars the associated uncertainty. The fit is shown as a full-drawn line. **b)** Proposed exchange process for cTAR DNA **2** with rates and populations derived from the relaxation dispersion curves in **a**.

Supporting Table S1. Comparison of proton chemical shifts of ground and excited states of RNA **1** and DNA **2** with reference data.

A-site RNA **1**

residue	δ (GS) / ppm 12°C	$\Delta\delta_{ES-GS}$/ ppm 12°C	δ (ES) +/- / ppm 12°C	ΔU95 mutant Δδ / ppm[9] 25°C	N^3M-U95 mutant Δδ / ppm[9] 25°C
C7	7.88	0.24	8.12/7.64	0.10	0.29
C9	7.42	0.15	7.57/7.27	0.10	0.06
A10	8.04	0.22	8.26/7.82	0.11	0.06
A92	8.02	0.14	8.16/7.88	0.16	0.19
A93	8.21	0.24	8.45/7.97	0.70	0.38
G94	7.31	0.34	7.65/6.97	0.45	0.14
U95	7.48	0.29	7.77/7.19	n.a.	0.25

n.a. not available

cTAR DNA **2**

residue	δ (GS) / ppm 25°C	$\Delta\delta_{ES-GS}$/ ppm 25°C	δ (ES) +/- / ppm 25°C	B-form DNA δ / ppm[10]
G4	7.88	0.25	8.13/7.63	8.1 - 7.3; maximum occurence: 7.9
A5	7.82	0.49	**8.31**/7.33	8.5 – 7.8; maximum occurrence: 8.2
C19	7.35	0.22	7.52/7.13	7.8 – 7.0; maximum occurrence: 7.3-7.4
G20	8.14	0.20	8.34/**7.94**	8.1 – 7.3; maximum occurrence: 7.9
A21	7.87	0.56	**8.43**/7.31	8.5 – 7.8; maximum occurrence: 8.2
C22	7.72	0.34	8.06/**7.38**	7.8 – 7.0; maximum occurrence: 7.3-7.4
C23	7.87	0.24	8.11/**7.63**	7.8 – 7.0; maximum occurrence: 7.3-7.4

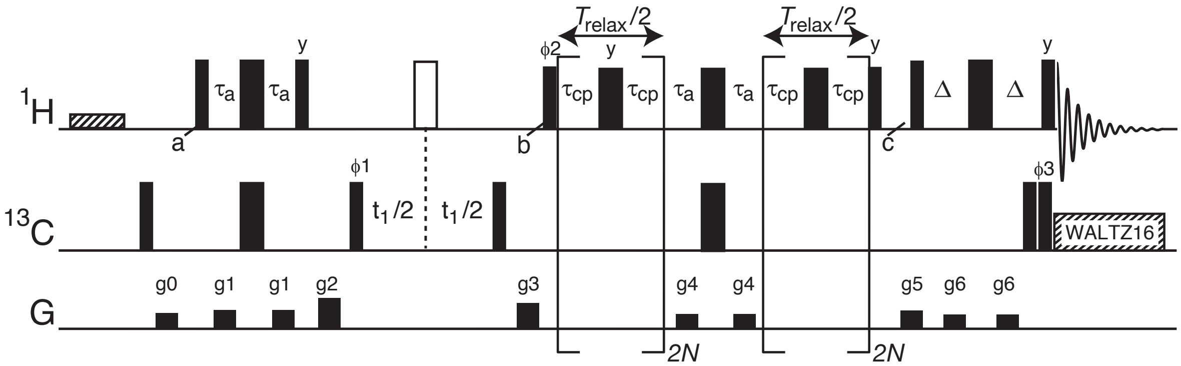

Supporting Figure 5. Pulse scheme of the RNA and DNA H6/H8 constant-time relaxation dispersion experiment for measuring millisecond time-scale dynamics of 6-^{13}C-5-D-pyrimidine and 8-^{13}C-purine labeled nucleic acids. The pulse scheme follows closely the scheme of Ishima et al. to measure millisecond time-scale dynamics of amide protons in per-deuterated proteins.[11] ^{1}H, and ^{13}C 90° (180°) rf pulses are shown as narrow (wide) black bars. The hashed ^{1}H pulse represents a pre-saturation of the H_2O magnetization and the ^{1}H 180° pulse during ^{13}C chemical shift evolution (open box) is given as a composite 180° pulse: $90_x180_y90_x$. ^{1}H and ^{13}C pulses are applied at the highest possible power level, except between *b* and *c* where the power of the proton pulses is reduced by 3 dB. All pulse phases are assumed to be x, unless indicated otherwise. The ^{1}H carrier is placed on the water signal at *a* and moved to the middle of the H6/H8 region (7.6 ppm) during the CPMG element between *b* and *c*, whereas the ^{13}C carriers is in the middle of the C6/C8 region (140 ppm). ^{13}C decoupling during acquisition is achieved with a WALTZ-16 scheme applied at a field of 2.3 kHz.[12] The phase cycling used is: ϕ1 = {x -x}, ϕ2={x x -x -x}, ϕ3={x x x x -x –x -x -x}, and receiver={x -x -x x}. The delays used are $\tau_a = 1/(4J_{CH}) \sim 1.18$ ms and Δ = 0.8 ms. *N* is any whole number. Gradient strengths G/cm (length in ms) are: g0=1(0.75), g1=3.5(0.75), g2=25(0.75), g3=17.5(0.75), g4= 6.5(0.75), g5= 11.5(0.75), g6=4.5(0.5). Quadrature detection in the indirect dimension is obtained via State-TPPI of ϕ1.[13] The part of the pulse scheme between *c* and *acquisition* can be replaced with a single ^{1}H 90_x pulse at the expense of slightly worse water suppression.

Supporting References

1. Wunderlich, C.H., et al., *Synthesis of (6-13C)Pyrimidine Nucleotides as Spin-Labels for RNA Dynamics.* Journal of the American Chemical Society, 2012. **134**(17): p. 7558-7569.
2. Delaglio, F., et al., *NMRPipe: a multidimensional spectral processing system based on UNIX pipes.* J Biomol NMR, 1995. **6**(3): p. 277-93.
3. Hansen, D. http://www.biochem.ucl.ac.uk/hansen/fuda/.
4. Hansen, D.F., P. Vallurupalli, and L. Kay, *Using relaxation dispersion NMR spectroscopy to determine structures of excited, invisible protein states.* Journal of Biomolecular NMR, 2008. **41**(3): p. 113-120.
5. Hansen, D. http://www.biochem.ucl.ac.uk/hansen/catia/.
6. Hansen, D.F., et al., *Probing Chemical Shifts of Invisible States of Proteins with Relaxation Dispersion NMR Spectroscopy: How Well Can We Do?* Journal of the American Chemical Society, 2008. **130**(8): p. 2667-2675.
7. McConnell, H.M., *Reaction Rates by Nuclear Magnetic Resonance.* The Journal of Chemical Physics, 1958. **28**(3): p. 430-431.
8. Press, W., et al., *Numerical Recipes*. 3rd ed. 1988.
9. Dethoff, E.A., et al., *Visualizing transient low-populated structures of RNA.* Nature, 2012. **491**(7426): p. 724-728.
10. Wijmenga, S.S., M.M.W. Mooren, and C.W. Hilbers, *NMR of Macromolecules - A Practical Approach*. The Practical Approach Series, ed. D. Rickwood and B.D. Hames. 1993: Oxford University Press.
11. Ishima, R. and D.A. Torchia, *Extending the range of amide proton relaxation dispersion experiments in proteins using a constant-time relaxation-compensated CPMG approach.* Journal of Biomolecular NMR, 2003. **25**(3): p. 243-248.
12. Shaka, A.J., et al., *An improved sequence for broadband decoupling: WALTZ-16.* Journal of Magnetic Resonance (1969), 1983. **52**(2): p. 335-338.
13. Marion, D., et al., *Rapid recording of 2D NMR spectra without phase cycling. Application to the study of hydrogen exchange in proteins.* Journal of Magnetic Resonance (1969), 1989. **85**(2): p. 393-399.
